# Exploring the life stories of young adult men in prison with a history of dual harm

**DOI:** 10.3389/fpsyt.2026.1829780

**Published:** 2026-05-20

**Authors:** Lindsay Thurston, Karen Slade, Nicholas Blagden, Thom Baguley, Luke P. Vinter

**Affiliations:** 1 Department of Health Sciences, Faculty of Sciences, University of York, York, United Kingdom; 2 Department of Psychology, School of Social Sciences, Nottingham Trent University, Nottingham, United Kingdom; 3 Department of Criminology, College of Health and Humanities, University of Derby, Derby, United Kingdom

**Keywords:** dual harm, narrative analysis, prison, qualitative, self-harm, violence

## Abstract

People who engage in both self-harm and violence (‘dual harm’) in prison cause widespread disruption to prison services. Whilst the behavioural profile of such individuals is gaining attention, there is very little research which explores their life histories and how these contextualise their dual harm. This study qualitatively explored how five young men in a medium secure prison in England with a history of dual harm (in the community, prison, or both) made sense of their life experiences and engagement in dual harm behaviours. Participants were interviewed using an in-depth life story interview protocol. A narrative analysis identified three themes: ‘Beginning: Making sense of a traumatic childhood’, ‘Middle: Exploring challenges during late adolescence’ and ‘End: Who I am now, and who I must be’. These themes, grounded in life experiences and associated meaning, offer valuable insights into the underpinnings of dual harm behaviours and direction for interventions addressing dual harm in prisons. The paper concludes with a discussion of key implications and directions for future research and practice in relation to the support and management of people who dual harm in custody.

## 1 Introduction

In England and Wales, young adult men aged 18–20 comprise less than 4% of the prison population (1). Despite this, they account for 17% of all male self-harm in prison (2) and have the highest rates of recorded violence across all age groups (3). Research that analysed nationally representative data from His Majesty’s Prison and Probation Service (HMPPS) indicated that 12% of young men in custody engaged in both self-harm and violence (4), referred to as “dual harm” hereafter (5, p. 98). Across judicial systems, people who dual harm exhibit elevated rates of fire-setting, property damage and disorder, spend longer in segregation, and engage in more severe self-harm (6–10). These characteristics demonstrate the complexity of people who dual harm and the challenging behaviours they display. Gaining a deeper understanding of the lived experience and development of dual harm, and areas of risk and need, can improve safety and wellbeing in prisons.

Although the behavioural profile of people who dual harm in prison has become clearer (9), less is known about their pre-prison life experiences and how these may contextualise dual harm behaviours. One qualitative study found that adult men who dual harmed in prison used both self-harm and violence to reject identities rooted in adverse childhood experiences (ACEs; 11). ACEs have been linked to emotion dysregulation, reflecting a potential lack of support to learn, identify or express emotions during childhood (12). This aligns with studies of sole self-harm, in which men in prison reported self-harming to cope with childhood trauma in the absence of learning how to express their feelings more adaptively (13, 14). ACEs, particularly being a witness or victim of violence, can also lead to the behaviour being normalised, as has been reported by people who are solely violent (15, 16) and those who dual harm (17). ACEs have not been directly explored with young adults in prison who dual harm, though the presence of problematic childhood relationships did not distinguish young adult men who dual harmed in prison from those who did not (4). Despite this, negative childhood experiences likely relate to dual harm through how they are subjectively experienced and understood. However, no studies have explored how young men in prison interpret their childhood experiences in relation to their later dual harm behaviours.

Further early-life disruptions have been identified which may provide context to the development of dual harm. People who dual harm in prison tend to have fewer qualifications (4), lower reading scores (10) and less educational development during imprisonment (9) than those who do not. However, young adult men were not more likely to report learning difficulties (4). Factors beyond inherent educational ability, such as low school bonding or disrupted educational progression, could therefore be relevant in the pathway to dual harm (18). Nationwide cohort data revealed that people with a history of self-harm before age 24 have higher school absences and exclusions than those without the history (19). School truancy can be conducive to establishing relationships with anti-social peers and influence engagement in violent and delinquent behaviours for acceptance (20). Such relationships can shape offending identities in young adulthood (20), likely strengthened by narratives linking masculinity to crime and violence (21). These pathways may explain why young adult men with a history of dual harm in prison often have earlier and more extensive contact with the criminal justice system (4), consistent with the ‘school-to-prison pipeline’ (a framework that links low educational attainment to early imprisonment; 22). However, little is known about how and whether these educational experiences and associated identities become qualitatively relevant in trajectories toward later dual harming behaviours.

Within prison, research indicates a range of functions for harmful behaviours. For example, it has been suggested that young men may exhibit violence to deter threats or victimisation (23, 24), whereas self-harm may be used to express and release anger in a way that is not met with punitive institutional responses (25). Interviews with other forensic populations have found that the choice between self-harm and violence is typically influenced by each behaviour’s perceived usefulness and accessibility (11, 17). It is not yet known whether these functions and the decision-making process hold the same relevance for the duality of harm within young adults in prison.

The present study explored the life stories of young men in prison who each self-reported a history of dual harm. It aimed to explore key experiences within their life stories, how these experiences were made sense of and narrated in relation to their dual harm behaviours, and identify thematic commonalities and differences across participants’ stories.

## 2 Methods

### 2.1 Ethics

A favourable opinion for the research was obtained from HMPPS National Research Committee and Nottingham Trent University’s Business, Law and Social Sciences Research Ethics Committee.

### 2.2 Setting

This study was conducted in a Category B prison in England which houses men aged 18+ serving less than 12 months in custody.

### 2.3 Participants

Posters were displayed on all wings inviting eligible individuals to express an interest in taking part in the study. Posters outlined the main aims of the study, eligibility criteria, and clearly stated that no rewards were offered in exchange for participation. Five men aged 18-21 (*M* = 19.60; *SD* = 1.14) responded to the posters with an expression of interest and all five participated in the study. Each participant self-reported a lifetime history of dual harm (as per their own definitions of self-harm and violence) which included behaviours exhibited in the community (i.e., pre-prison), in prison, or both. Participants understood self-harm to be a physical act of harm directed towards the self, regardless of method or suicidal intent. Violence was described as an intentional, physical act of harm directed towards others, and whilst threats of harm were discussed, all participants had perpetrated at least one act of physical violence. These definitions of self-harm and violence correspond to those routinely used in theoretical frameworks and research (26, 27). All participants had sufficient English language skills to take part in the interview and posed no risk to lone females. Official prison records reported participants’ ethnicities as being White British (*n* = 4) or Mixed, White and Black Caribbean (*n* = 1). Participants had spent between 47 and 246 days in prison during their current sentence.

### 2.4 Data collection

The researcher (lead author) met with potential participants in a private room within the prison’s education department. Participants read an information sheet (or had it read to them) stating what the research entailed, what their participation would involve, and how to withdraw both during and after the interview (either verbally to the researcher or via the prison application system). Participants were encouraged to ask questions about the research, and their involvement in it, before giving their full written consent.

Participants were asked to re-tell their life as if it were being made into a film and consider how it could be deconstructed into various scenes, each depicting an important life event or experience. Interview questions were guided by McAdams’ (28) ‘Life as a Book’ life story interview (LSI) protocol, though in keeping with arguments that people in prison may struggle to identify with the book concept, a film analogy was used (29). This analogy is considered more age and culturally appropriate and it is suggested that participants feel less obliged to justify their actions which allows for more fluent storytelling (29). Participants were asked to share key scenes, high points, low points, and their greatest challenges and influences (28). Each participant completed their LSI across 1–2 sessions, and in total, these lasted 75–199 minutes (*M* = 145.60; *SD* = 50.83). Interviews were recorded on a password-protected dictaphone and transcribed verbatim with identifying information being omitted. One participant only completed half the interview due to being released from prison on bail between sessions.

### 2.5 Data analysis

As narratives are a key means through which individuals attribute meaning to experiences and behaviours, particularly those which deviate from social norms (30, 31), a narrative analysis was conducted to understand how each participant “lived, experienced and interpreted” their life and dual harm trajectories (32, p. 45). Such approaches emphasise that by organising and linking together an individual’s past, present and anticipated future, narratives help to make sense of and find meanings in lived experiences (32–34). Throughout the life course, individuals collate these internal narratives and link them together to form a more coherent understanding of their life and sense of self (32, 35).

Whilst there is no prescribed method to conduct or analyse narrative research, the analysis in this study was guided by the works of Crossley (32) Langdridge (36) and Murray (37). First, transcripts were checked against the original recordings for accuracy, enabling for familiarisation of each transcript and initial notes or patterns between transcripts were made. Overall tone (i.e., how participants used emotions to construct their narratives) and rhetoric (i.e., whether participants constructed their narratives to distance, argue, justify, convince, or position themselves in a specific way) were identified. Identity work was also considered, which documented how each participant constructed their identity within the narrative. Informed by these steps, a narrative summary was created for each participant’s life, which followed a chronological timeline. Last, whilst not losing sight of the narratives being described, key themes were identified within and across participants’ stories. Here, the aim was not to reduce the text into disparate phrases or individual codes, but rather to capture prevalent thematic patterns in the data. Superordinate and subordinate themes grounded in the data were created and labelled, and pseudonyms were ascribed by the researcher to maintain participants’ anonymity.

### 2.6 Reflexivity

Narratives are not simply fixed accounts of an individual’s experiences but may also serve important social functions during the interactions in which they are shared (33). Therefore, it is important to acknowledge and reflect upon the influence of the researcher’s positionality in the construction of participants’ narratives. The lead researcher conducting the interviews in this study was a female academic who was of a similar age to the participants. Whilst this may have enhanced rapport, it is possible that it also influenced the framing of participants’ narratives. For example, themes relating to masculinity and impression management permeated throughout the narratives, which may have, in part, been a byproduct of the relational dynamics of this research-participant interaction. The lead researcher discussed these themes in reflective supervisions with the broader research team throughout data collection, and used such discussions to share emerging thoughts and insights. Furthermore, although the lead researcher had previous experience of working within a prison, for the purpose of the present study, she entered the prison and engaged with participants as an independent outsider. This unique positionality enabled meaningful exploration of participants’ experiences, whereby the researcher’s prior employment equipped them with sufficient familiarity of the prison context, whilst offering sufficient distance to enable participant openness. This was evidenced by one participant in particular, who had found the research process to be emotive yet cathartic. At the end of his interview, he remarked that he had felt able to share his life story so openly because he perceived the researcher to be a ‘nobody’.

## 3 Results

Within their LSI, participants made sense of their experiences by using story-like narrative patterns (32). Specifically, life stories were narrated with a beginning, middle and end formation (38), held together by plots, events and characters (34). Significant life events were linked to past experiences and relationships, ranging from childhood to teenage years, and concluded with the present day. Reflecting this, the analysis identified three superordinate themes (see **Table 1**).

**Table 1:** Superordinate and subordinate themes identified through narrative analysis.

Superordinate themes	Subordinate themes
1. Beginning: Making sense of a traumatic childhood	1.1. Turbulent family relationships1.2. Finding belonging in all the wrong places
2. Middle: Exploring challenges during late adolescence	2.1. Striving for agency and mastery 2.2. Intense relational experiences2.3. The self as a protector
3. End: Who I am now, and who I must be	3.1. Grappling with the present self 3.2. Expression and regulation

### 3.1 Superordinate theme 1: beginning: making sense of a traumatic childhood

Participants’ early life narratives were frequently characterised by a negative tone and a sense of turbulence, which contextualised the later chapters of their life stories. This tone was particularly noticeable in the descriptive detail used when recalling negative childhood memories, including ACEs. Participants also described how peer relationships were influential on their understandings of self and early engagement with self-harm, violence and other antisocial behaviours. Ultimately, although there was diversity across individual experiences, all participants positioned their childhood experiences as pivotal to their later identity formation, behaviours, and relationships.

#### 3.1.1 Subordinate theme 1.1: turbulent family relationships

Participants’ childhood narratives were rooted in stories of relationships. They frequently contrasted their desire to feel safe and cared for as a child against the harsh realities that they experienced, including experiences of intrafamilial abuse and neglect. In many cases, participants described situations where family members had failed in their roles as reliable caregivers and protectors, which had profound impacts on them and how they understood themselves in later life.

“My dad abused me. Before I got put in care my dad and my auntie both abused me and my sister, I got stabbed by my mother, I got my bedroom set on fire by my auntie, when I was still in the bed obviously, I got starved, I got left in the house on my own when I was just a baby … was covered in cuts, scars and bruises and had to have all my teeth taken out, which must explain why I look like a vampire now, but you know, roll with the punches [laughs].” (Shaun).

Here, Shaun details the severe and recurrent abuse he experienced at the hands of his family. Despite the horror of these early life experiences, Shaun quickly employs humour and a snap back to present day to diffuse the emotional charge of his narrative, deflecting conversation away from these harrowing experiences. Nevertheless, like other participants in this study, Shaun’s early life was characterised by a prevailing sense of threat and instability, with limited opportunities for sanctuary. In many participants’ early life narratives, the very people that would be expected to offer care and safety were those who represented threat and unpredictability. For example, Ethan, a secondary victim of intimate partner violence, described a sense of incoherence in his family dynamics:

“It was always my job to protect mum, it’s who I am … Telling my dad to not hit my mum. Probably that is the worst negative because that was horrible, and seeing my mum cry, screaming and that to then like, telling my dad to get off her and that, she used to scream ‘help’ as well like, ‘help, help me please, my kids are here please don’t do it to me’ and shit like that and then when mum was pregnant it all changed, changed him man … I felt happy, I felt like I finally had my family back again … he treated my mum like a fucking, you don’t even understand, he used to go, he used to do everything for her … I can picture it now, my mum was sat, like sat on the, like, leant against the side and my dad was on his knees kissing my mum’s belly.” (Ethan).

On one hand, in Ethan’s early life narratives, his father was positioned as a villain, leading him to adopt the role of hero and protector, an identity that re-emerges in later stages of his life story. Despite being young, he described intervening and protecting his mother and, by doing so, he characterised himself as a courageous child. The quote “*it’s who I am*” suggests that this protective role remains a stable part of his current narrative identity. On the other hand, Ethan fondly recalled how his father “*changed*” and would dote on his mother during her pregnancy, which re-established the family unit. Here, there was a notable contrast between Ethan’s negative experiences (witnessing intimate partner violence) against more idyllic family moments, further illustrating the overarching sense of turbulence that lays at the heart of this theme. Whilst there is thematic consistency across participants with regards to the presence of turmoil, abuse and neglect, participants did not always make sense of their experiences in identical ways. For example, where Ethan had portrayed his father as the villain, his mother as a victim, and himself as protector, Matty (below) ascribed some blame to his mother (also a victim of domestic abuse) for not shielding him from the traumatic violence he witnessed:

“I say it to my mum now that she was like partly to blame because if she walked out of that household I wouldn’t be, I wouldn’t have seen what I seen. I wouldn’t have seen my mum getting beat up and plates getting thrown in her face and stuff like that, so, that’s obviously when she finally got up and left … If I was to, if I hadn’t seen what I seen in that house, I wouldn’t be the way I am.” (Matty).

Matty positions himself as a helpless child who had been let down by his mother and exposed to scenes that could have been prevented. He uses this construal of his past experiences to make sense of his present-day identity, understanding himself as a product of the traumatic scenes that he had not been protected from. Here, Matty found meaning and coherence in relation to his experiences by creating a narrative of cause and effect; he made sense of his identity and behaviours by storying them as inevitable consequences of his early life experiences.

Although stories of pain and trauma were key features of participants’ early life experiences and relationships, in keeping with the notion of turbulence, participants also introduced opposing stories of love and compassion. Often, these stemmed from relationships with grandparents:

“I’ve looked up to my grandad as a dad, like, every time my mum argued with that partner of hers we’d go straight to my grandad’s and stay there for a couple of weeks and he’d sort everything out. He would just mould everything back together so everything was alright again … My grandad even took me to school. That’s the sort of stuff you should be doing with your mum and dad when you’re growing up. Learning to ride bikes and stuff but that’s what my grandad did. So that relationship with my grandad is more like a father. I looked up to me grandad because he was basically my dad … I tried killing myself when my grandad died … That person who used to put everything right, the person who used to put everything together, had gone. My family will never be together again, that won’t change, it won’t get better.” (Matty).

“I think I was about 10 or 11…. we used to have exact timelines of seeing her [grandmother], like my brother’s high school was right outside her house so we’d go there like every time, we’d stay there, have food, and then sometimes it would just hit me, that she weren’t there. I just wanted to take the pain out, so then I just tried taking it out on myself really, I like, I started cutting on my arms. Nothing bad, like, my mum didn’t even notice once, or my stepdad, and if they did know they’d have gone mad at me, so I know they definitely didn’t.” (Ben).

In the first extract above, we see Matty’s grandfather represented as a figure of refuge and security, whom he idolised as a role model, taking on the qualities, responsibilities and actions that were made not available from his parents. Similar feelings were expressed by Ben in relation to his grandmother, a figure who was characterised as a nurturing source of care and emotional warmth, in contrast to his more distant mother and stepfather. Both Ben and Matty constructed a narrative whereby their self-harm and suicidal behaviours were triggered and explained by the loss of their beloved grandparent. For Ben, cutting his arms was described as a way of coping with the intense pain he experienced at the loss of his grandmother. This may signify that owing to Ben’s young age and his childhood experiences, he may have struggled to regulate his emotions effectively. For Matty, his grandfather had been a redeeming source of light in an otherwise extremely adverse childhood and offered the strongest sense of attachment that he had experienced in that time of his life. The profound sense of loss that he had experienced upon his grandfather’s death had seemingly extinguished his hope to find said light again, contributing to his suicide attempt.

This theme illustrates how experiences of family relationships, good and bad, were fundamental to participants’ early identity formation and sense-making of their present selves. We also see examples of how relational experiences bear relevance to acts of self-harm, a pattern that reappears in later life story chapters too.

#### 3.1.2 Subordinate theme 1.2: finding belonging in all the wrong places

This subtheme captures participants’ descriptions of early peer relationships, and how they became relevant to self-harm, violence, and other forms of antisocial behaviour. Here, participants emphasised the value of these relationships, and how antisocial behaviour was sometimes displayed to establish or maintain relationships with peers. Moreover, participants frequently described how childhood social groups and peer role models would influence and reinforce these behaviours. For example, Brendon ascribed some of his earliest offending behaviours to an older set of friends from the “*wrong crowd*”, who continued to entice him to engage in drug dealing:

“I got involved in the wrong crowd of people from the estate that we used to live on … All the older lot who I got in with, they were the wrong crowd of people, wrong kind of people … I got involved with weed from the older people. All the drugs I were selling at one point was just weed and then they asked me if I wanted to start making some extra money” (Brendon).

However, beyond material benefits, at the heart of participants’ narratives was a desire for communion with others. Whilst participants frequently ascribed some responsibility for their antisociality to the influence of peers, it appeared that engaging in these behaviours often functioned as a means to establish a sense of belonging and social connectedness. For example, as a child, Ethan acquired a reputation as someone who was both fearless and someone to be feared. Despite facing social disconnectedness and exclusion from some due to this reputation (e.g., prosocial peers), he found more antisocially inclined peers who seemingly appreciated and reinforced these qualities:

“Friends who I hung around with as a kid, and if you have negative people around you then you’re likely to have negative attention. I was that person yeah, like, everyone was scared of me as a kid yeah, everyone was … but everyone was scared of me miss because I, I will do it init. Like if someone told me to do summats I will do it init. Like if someone told me to go down the hill in the trolley, I would do it … But I like who I am and I wouldn’t be who I am if I didn’t have them friends around me, I’m not a bad, I’m not a bad, I wouldn’t say I was a bad person.” (Ethan).

Ethan’s storying suggests he may have maintained his troublesome behaviours to seek acceptance and belonging from the “*negative people”* he described. He reported feeling coaxed by these friends into ‘acting up’ during school, which served to maintain his connection and belonging with them. However, in doing so, this simultaneously led to his school suspension, and implicit exclusion from opportunities for prosocial connections. It is possible that, over time, Ethan found a greater sense of belonging, acceptance and a coherent self-identity in groups that reinforced antisocial norms, and a sense of disconnection with groups that model prosocial norms, which perhaps underpinned subsequent deviance. That said, there was evidence of subtle conflict in Ethan’s narrative, despite recognising these early friendships as having a negative influence on his behaviours, Ethan appreciated his friends for having a positive influence on shaping him into person he has become, a version of self that he feels confident in. It is possible that this apparent conflict is a consequence of later life reflections, where Ethan has been able to retrospectively dissect his early life and identity formation in a nuanced way, recognising positive and negative facets of his early relationships beyond a more binary categorisation. Nonetheless, Ethan’s framing of antisocial behaviour as a key to acquiring an early sense of social connection and belonging resonated across participants and extended beyond general criminality to violent and self-harming behaviours. For example, Ben became a member of a gang by his 14^th^ birthday, and described how his membership in the gang was predicated on engaging in violence:

“You’re all like friends in the gang, and like my friend has trouble with him so now we’ve all got trouble with him, and in the end you just end up getting all sucked together, then yous are all like just there really. In the gang you soon start playing with like guns, knives, trying to stab people, beating people up … We protect each other. It’s what we do in the group. We all claim to protect each other. We all claim it so we have to show that we mean it and that we have loyalties to each other. I don’t like expect anybody to go out and do something for me, but, when we have that like, family vibe with each other, or say like something happens, then, I know I’ve got you and I kinda expect you to have me.” (Ben).

For Ben, violence served as a necessary facet of gang membership, to maintain his status as a member of the gang and adhere to norms of reciprocity with peers. Ben suggested that membership with the gang provided a sense of belonging, purpose and, importantly, safety that could not be found elsewhere. Themes of protection and loyalty in the face of shared adversity weaved through Ben’s narratives about his gang membership. Ben implied that the “*family vibe*” of the gang naturally elicited reciprocal support, which vastly differed to the support and protection he experienced from his mother during childhood. This resonates with theme 1.1 and suggests that the desire for safety and social belonging can be fundamental needs for humans. Resonating with Ben’s description of violence as a necessary evil for maintaining a place in a social group, Shaun described how, as a teenager, he had utilised performative self-harm to develop relationships and social standing with peers:

“I was about 14 when I started [self-harming] … I was kinda at an Emo stage with two of my mates … everyone seemed to be doing it really, just like cutting wrists and that, nothing particularly bad, it just looked cool, but I thought I’d take it one further, so I just fully stuck a kitchen blade into my arm … Yeah, it was good. My friends who were there at the time couldn’t believe what I’d done, so that was pretty good. The birds thought I was well hard too because I didn’t even feel anything from it, so yeah, it was decent.” (Shaun).

Shaun framed his earliest acts of self-harm as instrumental to fit in with his peers and the ideals of the broader “*Emo*” subculture that they associated with. For Shaun, feelings and emotions preceding acts of self-harm at this point in his life were largely absent. Instead, he primarily used his recollection of self-harm as a teenager to share how his peers positively endorsed the behaviour, and how the behaviour reinforced to others a version of self that he was happy to project (i.e., someone who was brave, fearless, and felt no pain). This sense of approval and reinforcement from peers resonates with other participants’ narratives around the functions of their violence, self-harm, and/or antisociality in early life. As children, participants discovered that, within the context of certain social groups, acts of violence and/or self-harm could function as a relational tool for the enhancement of social connection and status. External reinforcement of these behaviours from peers could then serve to further entrench this perceived function, perhaps representing an important early stage of participants’ trajectories toward dual harm in later life.

However, whilst this notion of engaging in harmful behaviours to relate to others permeated through all participants’ early life stories, there were limits. This is abundantly clear in Matty’s life story. Similar to Brendon, Matty associated with older peers as a child, and engaged in criminality (including violence) to appease those individuals who he had initially respected as role models to maintain his place in their group. Akin to Ethan, Matty was also excluded from mainstream education and attended a pupil referral unit, finding opportunities for belonging as part of this deviant social group. However, Matty described limits to what he was prepared to do to be part of this group, recalling a nadir point in his life story:

“I was only about 10 or 11 and meeting up with 16-year-olds. I’d just tag along and they’d just buzz off me because I used to just terrorise everyone, I was just an idiot really, like if there was a bike parked outside the shop I just used to pinch it for fun, I didn’t even want it … one day though, I seen summats what I shouldn’t have, that I didn’t want to see. They kicked a door off and beat a man up. I ran down the stairs me and took off, I was young, and they did summats to that man that they went to jail for. They put a sky remote up his arse and that and I didn’t see that, I just saw them kick the door down and beat fuck out of him but because they were vallied up out of their heads, and I was scared then, I was fucking terrified, because I’d never seen them be like that. I ran, I just kept on running and running and running until I couldn’t see them again … Stuff like that ruins peoples’ lives. It can ruin lives, it’s the worst ever thing you could do to someone. I just went mad after all that, because I didn’t have anyone to look up to no more because I’d looked up to fucking sex offenders. But I’m not a sex offender right miss, I’m not, I’m not just saying that for the tape.” (Matty).

When storying the incident of sexual violence, Matty’s narrative tone and position shifted, he repositioned himself from a proud troublemaker as part of an established social group to a scared and alone young man. Matty recognised sexual crime as a step too far and firmly distances himself from both the older peer group and their behaviours. To this end, Matty makes concerted efforts in his narrative to represent himself as qualitatively different to those who perpetrate sexual offences. In his telling of this event, Matty conveys a sense that his worldview had been utterly shattered. With his main social role models and relationships gone in an instant, Matty was seemingly lost, without role models, relationships or a coherent sense of self to fill the void, serving as a destabilising turning point for him thereafter (“*I just went mad after all that*”). Matty’s experience illustrates that influences of early peer relationships and group memberships on harmful behaviours can be complex and nuanced. Whilst a desire to belong to a social group can underpin some deviant behaviours and the formation of antisocial identities, this process is not black-and-white, and existing internal mechanisms and entrenched values can protect against harmful behaviours in the face of external pressures.

### 3.2 Superordinate theme 2: middle: exploring challenges during late adolescence

This theme proceeds from participants’ childhood narratives to mid-late adolescence (age 14-19). In this theme, participants narrated shifts in their family lives, social groups and intimate relationships. This shift was also accompanied by the development of multiple and dissonant identities, which subsequently related to their self-harm and violent behaviours.

#### 3.2.1 Subordinate theme 2.1: striving for agency and mastery

Desires for agency, autonomy, individuality and mastery became key features in participants’ life stories as they described their formative transitions through mid to late adolescence. For example, leaving home and breaking free from family units were often described as important life events, allowing participants to act more freely and discover a sense of independence:

“I had my own flat at one point with [housing provider], when I was about 17 or 18, but I ended up losing it for a bladed article … It was good though, I’d rather live on my own. I’d rather have my own place than live with my mum because it’s time to step up init? You have to know what you’re doing then.” (Brendon).

Brendon saw the move out of his family home as representing an opportunity to establish independence and master his life. Whilst for others, agency was centred around being their own person and correcting a dissonance between the person they were encouraged to be and the person they understood themselves to be, for Brendon, agency was achieved through being able to “*step up*” and take responsibility.

Other participants described ways that more harmful behaviours, such as violence, had been utilised to regain a sense of agentic control over their lives. For Ben, this took the form of physically fighting with his stepfather:

“He [stepdad] tried to grab me by like my neck and he’s like pinning me down on the sofa and I kept saying, I said to him like twice, let go because when I do get hold of you I’m gonna punch you, but like, he wasn’t listening … at the back of the sofa I had these like carbon gloves, like motorbike gloves, and I just remember I put them on and hit him twice and I just remember him like falling onto the sofa … So, in the whole, wider situation, like when he’d rag me about, I just felt like I won.” (Ben).

Ben had portrayed his stepfather as a villain and disempowering force in his early life story. However, Ben describes the first time he physically stands up to his stepfather as an adolescent, framing it as a redeeming triumph. It appeared that, For Ben, physical violence became a means to re-empower himself, to counteract the years of disempowerment he had experienced as a child at the hands of his stepfather. Violence became a way of expressing agency, demonstrating power and reclaiming control of the external world when under threat, which is evidenced in later episodes of Ben’s life story:

“I knew all these kids in my area and they wear knives like and go around together, and I don’t know, I went through a phase where I wanted to be like that … Now everyone in the circle, they all know, they all look to up me like I’m the main guy and I have been for years. Still to this day they look up to me like I’m the main guy … he [fellow gang member] said he had done this and that, and that he was gonna stab me in my neck, and I wanted to show people that he isn’t what he makes out to be … so I went downstairs, I got like a kitchen knife and I started sticking it in his leg and then I started stomping on him … I just wanted to like, put him down a bit and show others that he isn’t as big as what he makes out, because I knew he wasn’t but nobody else did, and I wanted them to see all that.” (Ben).

This extract highlights the progression from Ben wanting to join a gang to becoming a leading member, and how notions of violence and power become relevant to this shift. Whilst Ben had previously prioritised the sense of belonging and loyalty that the gang had provided him (see subtheme 1.2), his later narratives were centred around power and status. In this construction, he recalled stabbing a fellow gang member who tried to compromise his position. Ben enjoyed the prestige of being the “*main guy”* in the gang and became violent when his position was threatened. This showcase of violence allowed Ben to convince others of his power as “*the main guy*”, whilst reinforcing to himself that he has mastery over his environment and agency in his life. In addition to projecting himself as agentic and powerful, Ben’s narrative identity was also centred around authenticity. Ben felt the need to showcase his rival as a fraud, and not someone to be admired, whilst proving that he, himself, was the ‘real deal’. This implies that, beyond the acquisition of power, violence also represented a means for Ben to maintain an authentic expression of self.

Overall, this subtheme illustrates how desires for agency and power have been important forces in the lives of these young men and can act as powerful motivators for their decisions and behaviours. This is particularly important where these individuals have experienced childhoods often characterised by disempowerment at the hands of others, and perceived losses of agency.

#### 3.2.2 Subordinate theme 2.2: intense relational experiences

Relationships were key features of participants’ adolescent life story chapters, particularly romantic relationships. Frequently, participants described how they had tendencies to quickly develop deeply intense feelings of affection and idolisation towards romantic partners. They often idealised qualities of these relationships (e.g., security) and contrasted these against the turbulent relational experiences they experienced or witnessed as children. However, whilst these romantic relationships were held in such high regard in participants’ stories, they sometimes also served as antecedents to acts of self-harm and violence, particularly where they became associated with disruption or loss. For example, Ben described experiencing overwhelmingly intense feelings of love towards his first romantic partner:

“I was just too in love, I fell so deep in love with her, too in love and too soon. Then like, this one time I just felt like I’d had enough of it, enough of life in general really … my mum and sister didn’t really like me no more, no one really did, and I was arguing with my girlfriend and that, and it just instantly just started bringing stuff into my head like what’s the point of me even being here anymore?… We had this top light window thing with like this white thing going around it and I tied this thing around it and I like put it around my neck, like tied it to my neck and I just had all of this aggression, and like, hate to myself, and it was running through my body but then for like for some reason, after it all I just started to calm down, I felt much calmer, I realised there’s a lot more to life than this.” (Ben).

Ben seemingly struggled to reconcile his intense feelings of affection towards his girlfriend with experiences of friction and toxicity in the relationship, and a lack of connectedness with others in his life. Ben’s experiences of dysregulation in response to this internal dissonance and his inability to cope seemed to underpin a state of existential crisis (“*what’s the point of me even being here anymore?”)* and the subsequent incidence of self-harm that he described. This situation resonates with Ben’s earlier life experiences of loss, during which he self-harmed to ‘take out’ intense pain. Ben may have questioned his existence because he anticipated the loss of his girlfriend, feeling like a failure, perhaps believing that *he* was the main reason why the relationship was not working. Evidence of self-directed aggression and self-hatred is also apparent in the extract, which may have further hindered Ben’s ability to seek help, believing himself to be the common denominator in the relationship difficulties he experienced. These feelings of isolation and a perceived lack of social support may have therefore been relevant to Ben’s plan to tighten the ligature.

Other participants described similar maladaptive, harmful responses to relationship-related loss. For example, Shaun’s girlfriend sadly passed away when he was 17, which he identified as his ‘greatest challenge’ in life. Relatedly, at 18, Ethan described how the ‘high point’ in his life, learning that he was going to be a father, quickly became his ‘low point’ as he and his partner experienced a miscarriage. In response to their respective losses, both men engaged in violent and self-destructive behaviours:

“I just went on a bender, went on a spree basically … it was underground fighting … I was at such a low point of my life, I had no feelings, no emotions, I had no one at that point, I didn’t really care … I was letting my anger out, though even though I was letting my anger out it didn’t help, it didn’t change anything. I just didn’t have any feelings. Fighting didn’t even spark feelings in me, I was still just numb. I’d just lost her so I didn’t have anything, I didn’t feel nothing, I had nothing to lose did I, because I’d lost everything … I fell head over heels straight away, I seem to go with my heart over my head, because basically, shit that happened when I was younger, obviously I may have attachment issues.” (Shaun).

“I just felt so happy like, I don’t know, I just felt myself, I’ve never felt like that before. And then when I lost my baby, I just felt, I don’t know man … I just wanted it all to go away, I feel like that’s all led to it, like, all the anti-social behaviour and shit like that … because that was when I started to go off, like, off the rails, a bit aggressive and that, more violent, if anybody said owt I was just reacting.” (Ethan).

Both participants anchored their stories of violence within broader narratives of intimate relationships and loss. For Shaun, violence was a means to break through the sense of emotional numbness that he experienced. Whereas Ethan, utterly overwhelmed by intense negative emotions associated with the loss of his unborn baby, became perpetually dysregulated and violently reactive. Both participants had trouble articulating or labelling the exact emotions they felt during these periods, perhaps representing the difficulties they experienced processing said emotions in-moment. For example, Shaun refers to conflicting feelings of numbness and anger, and Ethan describes his feelings in very broad terms (“*I just wanted it all to go away*”). In any case, both participants seemingly defaulted to violence as a response to the overwhelming external and internal challenges that they had experienced.

Overall, all participants often implicated relationship hardships, breakdowns, and losses as key contextual factors in relation to their acts of self-harm and violence during their adolescent years. Most frequently these represented maladaptive responses to feelings of intense emotional dysregulation, a maladaptive means to regulate themselves or escape the difficult feelings. However, for some participants, acts of self-harm were also expressions of aggression and hatred towards themselves. This was true of Ben in his extract earlier in this theme, and was also true of Brendon:

“My mum phoned the police on me once because we were arguing all of the time about me getting into debt with other people, big people who then kept ringing my mum up … And the police put cuffs on me but said they weren’t gonna lock me up and that they were just gonna take me out of the house a minute, so then I sliced my wrist and they ended up locking me up. (Interviewer: How come you cut your wrist)?. Because I’d realised everything I’d done and I deserved it.” (Brendon).

Following relationship tensions with his mum and a related interaction with the police, Brendon suggests that he self-harmed as a form of self-punishment in response to feelings of guilt. It is possible that Brendon was cutting his wrist to atone for his perceived wrongdoings and to cope with the sense of guilt he felt toward his mother. Equally, it’s possible that, much like other participants in this theme, Brendon felt overwhelmed once confronted by the reality that his mum had called the police on him, and his self-harm was a maladaptive self-regulation strategy to manage those feelings, with the atonement narrative representing his retrospective attempts to establish narrative order and coherence. Nevertheless, emotional responses to difficulties within a close relationship continue to emerge as an underpinning feature of harmful behaviours.

#### 3.2.3 Subordinate theme 2.3: the self as a protector

Participants also began to present themselves as guardians, storying their violent actions as essential, predictable, and necessary to protect others. This often related to the protection of family and loved ones (e.g., partners) but could also include others they felt a sense of responsibility for (e.g., peers).

“Basically, I ended up scrapping with him [daughter’s stepfather] and beating 7 bells of shit out of him and I basically rang her [ex-girlfriend] up while he were there, video call and all that, and I were like is this the guy you’re getting to try and replace me? Is this the guy you want to be dad to my kid? Love, he can’t even protect himself, let alone protect you two. Basically, I wanted to teach him a lesson, to prove to her that yeah, I was better than him … coz basically that’s the dad’s job, to protect and provide for her, and if he couldn’t even protect himself what the fuck is he gonna do for a three-year-old kid and a girl.” (Shaun).

“The man tried hitting my Mrs. Orrr, listen, I swear to ya, awh the bang I gave him miss, he was snoring, he was snoring [laughs], he was like this in the flat [makes snoring noise] he was snoring. I hit him, I give it him, I couldn’t not. Just one chin shot, right on the end of his chin … But like, you see, you see that, I only done that because he actually went for my Mrs. If he didn’t go for my Mrs and only said fuck off to my Mrs, I might have been able to control it.” (Ethan).

Here, both men story their violence as acts of defence and protection of others, which became a familiar feature across all participants’ life stories. For Ethan, his act of retaliating against the individual who had harmed his girlfriend was a reactive form of defence and vengeance, which resonates with his early life narrative of standing up against his stepfather to protect his mother as a child (see subtheme 1.1). Shaun, however, appeared to view his actions more as proactive protection; proving that the stepfather figure was not competent enough to protect his daughter and ex-girlfriend, whilst reaffirming that he (Shaun) *could* offer what was needed. Whilst details and context differ, there is a consistency in the adoption of a guardian role, representing an underlying belief that violence is a valid and effective way to provide safety and security to loved ones. This sense-making also arguably serves to neutralise and justify their violence. This allows the men to make sense of their behaviours in a way that had positive implications for their identity. That is, they are ‘good’ people who protect those who cannot protect themselves. A sense of hegemonic masculinity is also implicitly woven into these narratives. In Shaun’s extract, there is a need to demonstrate (or ‘prove’) his dominant strength over the man he viewed as replacing him. Similarly, in Ethan’s extract, he weaves in boasting (“*just one chin shot*”) and humour to present a version of self that resonates with traditional conceptualisations of masculinity (i.e., a physically capable fighter).

Some acts of violence shared by participants represented more symbolic forms of protection, rather than literal. In the following extract, Matty recalls an incident during which an intruder was found rummaging through his grandfather’s belongings:

“There was a smack head outside of prison, I caught him in my grandad’s shed after he had died. He was trying to take lawn mowers, grass cutters and little antiques that my grandad would’ve worked hard for. If there was dust from when my grandad was alive, I wouldn’t even clean it you know because it was there when he was, I would never change it. He crossed the line when he went into that shed because everything in that shed was my grandad’s, it disrespected my grandad and all the family, so I smashed his head in with a spade and put him in hospital.” (Matty).

Matty understood violence as being deserved when someone seemingly disrespected the memory of his late grandfather. Here, we see the profound importance and admiration that Matty directs towards his grandfather, and how perceived threats to his memory warrant violent recourse. Beyond protecting his grandfather’s honour, Matty implies that his act of violence protected his whole family. This further reinforces his self-identity as being a protector, whilst also potentially serving to further justify the necessity of his violence, much like the neutralising narratives employed by Shaun and Ethan earlier in this theme. It also further illustrates the depth of meaning and importance that participants in this study attributed to key positive relationships in their lives.

### 3.3 Superordinate theme 3: end: who I am now, and who I must be

The final theme concerns participants’ present circumstances, during which they grappled with their identity in prison, and how this becomes relevant to dual harm behaviours. This theme is underpinned by a sense of survival and responding to the demands of prison.

#### 3.3.1 Subordinate theme 3.1: grappling with the present self

When narrating their current life scenes living in prison, the men revealed narratives indicative of a selfhood in turmoil. Present selves were questioned, compared with old selves, or temporarily reconstructed, leading to three identity-related patterns: existential crisis, reconciling the loss of former self, and developing an adaptive self to meet the environment’s needs. In the extract below, Shaun evidences an existential crisis of self, grieving the loss of his former life and questioning his life’s fundamental meaning and purpose upon entering prison:

“I don’t really think I can manage anything whilst I’m in here, because, they ripped me away from the whole set up I had in my life, they’ve stripped me of everything I had in my life. I’ve lost everything I had because of me being in here, I don’t even know who I am anymore.” (Shaun).

Shaun embedded his existential crisis in the emotional turbulence of being incarcerated and a sense of hopelessness. Feeling stripped of his former life upon entering prison and powerless to change or cope with his situation, Shaun expressed a profound sense of loss and identity crisis. Shaun expresses a fundamental lack of agency here, conveying a sense of emptiness and despair. He repeatedly refers to “*they*”, the criminal justice system, as those responsible for this, positioning the system as a villain in his story. By attributing his crisis to an external source, he attributes blame to an injustice perpetrated by the system more broadly, rather than accepting imprisonment as a consequence of his offending (which he denied).

Reflecting on their present-day identities in prison, other participants struggled to reconcile a sense of tension and dissonance between holding onto their former identities and accepting newer aspects of their identities that had developed since being in prison. For example, in the following extract, Ethan compares his past self (a wealthy provider), to his present self (a “*loner*”, a “*nobody*”).

“Prison. Jail, jail is the biggest failure, definitely. Errr, because, it’s not a good place. You can’t look after your family, you can’t provide for people, it’s a loner init. I’m a loner in here. Like, like a nobody. Anybody in prison is a nobody you know … Like if you come to, see me out there yeah, I would’ve never of thought that I would come to prison, I have money, I have, I have cars, I have motorbikes, I do things, I would never think I would come to prison with the things I do. I do good things. I like, I go sauna’s and that, I go gym, I do nice things.” (Ethan).

Here, Ethan compares materialistic differences in his life to illustrate how his circumstances, and, by extension, his identity had changed since coming into prison. The references to losses of money and other material possessions are used to illustrate a deeper sense of loss and unwanted change in himself. Interestingly, Ethan used the present tense (“*I have*”, “*I do*” and “*I go”)* to describe his lifestyle which may suggest that during the interview, he was still struggling to make sense of or accept the incoherence of his current identity and its inconsistency with his past self.

Brendon experienced a different identity shift. Less concerned with the discrepancies between his past and current self, he focussed on adopting a temporary survival-oriented identity in prison to meet the needs of the environment. This adaptive identity needed to respond to the social demands of prison life, and as such, Brendon crafted an intensified version of himself which centred around strength and masculinity. As part of this identity, extending the notion of violence as holding a protective function (see subtheme 2.3), Brendon construed violence as necessary to protect himself physically and socially. In the following extract, Brendon described a situation where he employed violence to clear an unmanageable debt, to preserve his social status in the prison, and to protect himself from future victimisation:

“I owed them [fellow prisoner] money so we just had a fight in the toilet, a one to one in the showers to clear the debt. It was them first, they started the fight with me, but I had to fight back to clear the debt because I couldn’t pay otherwise. I didn’t have the money to pay or whatever … I had to fight back otherwise he’d have battered me, I’d have looked a right dick and more people would’ve tried battering me probably because I’d look like an easy target”. (Brendon).

In this narrative, Brendon identifies as a ‘fighter’ who can contend with the demands of the prison social environment, rather than a ‘victim’. He emphasises the necessity of his actions, justifying the violence as an appropriate survival strategy. It is possible that, unlike other participants, Brendon does not experience the same conflict of identity in the prison, because the need to survive has been a facet of his identity throughout his life before prison (i.e., there is not as much of a disconnect, compared to the more materialistic changes cited by other participants). Similarly, Matty also described the adaptive version of self that he needed to adopt whilst living in custody, and how this, at times, necessitated violence:

“When I was in another jail I got done in, fucked up differently in my pad by a load of [county] lads. Beat up on a proper different level just because I was out of area … they put me back onto the same wing I come off, I split the lad’s head up, the same kid who did me over before, I did it with a chair leg. I just smashed his head in, but I did that because he took the piss out of me. Now he’s going to look into the mirror and see a scar on his fucking forehead and realise I did that, just like when I look into the mirror and see that he did that to me [points to own scar]. Then, when a lad from [county] landed in our prison I kettled him and he said ‘what was that for’ so I said, your lot got me when I was in [county], so I’m getting you while you’re in here. It wasn’t even the same guy it was some other kid, I shouldn’t have done it, I know I shouldn’t have done it, but it gets like that dunnit? I just reacted like that because others have done it to me.” (Matty).

Here, Matty not only describes how his pre-prison regional identity remains relevant in prison, but, like Brendon, positions violence as serving an adaptive function in a hostile, competitive environment. Matty acknowledges that his acts of violence served as a form of revenge, but also alludes to a belief that, in prison, violence could redress the reputational damage that he felt he may have suffered at the hands of his rivals. Rather than solely positioning himself as a victim, Matty constructed himself as masculine, physically powerful and capable in his response. Similar to Brendon’s sense-making of his violence in prison, Matty storied his behaviour as a way to survive in a social environment characterised by violence and competition. Matty’s narrative suggests that he considered himself to be ‘equal’ to the initial perpetrator (after leaving him scarred), and that the second act of violence [i.e., where he *“kettled”* (threw boiling water over) a perceived rival] enabled him to feel as though he had reasserted justice and protected himself against future attacks.

#### 3.3.2 Subordinate theme 3.2: expression and regulation

Participants often construed incidents of custodial self-harm as fulfilling one of two functions. On one hand, it served as a necessary means to communicate participants’ needs to the prison in a way that elicits a response from staff. On the other hand, it was described as a desperate, albeit private, act to cope with overwhelming emotions in a way that does not threaten image or reputation with peers. For example, Ethan described self-harming for the first time in custody, during which he cut his neck with a razor blade to prompt the prison to respond to his needs:

“You have to be on an ACCT to get seen. People who smash up and cut up and that, they get what they want. They get what they want every time. You want a telly or summats, cut your arm, you get a telly … because when you like ask them nicely they don’t want to do shit … But when I did that, they did it straight away. I was fuming because I’m trying to say to them yeah, why the fuck do people have to do this for you to pull a foot out or pull a thumb out your arse? It worked. I got what I wanted. But I shouldn’t have had to gone down that route because I asked nicely in the first place. (Interviewer: Can you recall why you self-harmed)? I swear they let me out late, they let me out late for my meds or summats. I think it was, or they took me off my meds. Summats, awh man, I can’t remember what it was. It was one of the two anyway.” (Ethan).

Here, Ethan suggests that his act of self-harm was a last resort effort to be heard by the prison, prompting the prison to initiate Assessment, Care in Custody and Teamwork (ACCT) procedures. Ethan implies that self-harm is a known pre-requisite to unlock in-prison privileges and to prompt action from the prison. In his narrative, Ethan frustratedly positions the prison as an apathetic and negligent figure that will only respond to the most extreme forms of communication and will ignore other expressions of needs. Extending the notion of survival and adaptive identities discussed in subtheme 3.1, it is possible that Ethan (and others like him) believe that self-harm can serve as an effective method to meet their support needs in prison and develop a greater sense of mastery in their environment. This could be particularly important for the likes of Ethan, who expressed a sense of struggle in adapting to a new identity and loss of a former self.

Beyond this communicative function, acts of self-harm were often described by participants as a way to cope with emotional turmoil. In an environment where there is a perceived pressure to convey an image of toughness and resilience to peers, expressions of emotion can feel exposing and induce a sense of vulnerability. To adapt to this, some participants utilise acts of self-harm behind their cell doors to privately express and release emotional overwhelm:

“I self-harmed a few times … I wouldn’t come out of my pad … I didn’t want others seeing me like that. (Interviewer: How do you feel in the lead up to self-harm)? I feel like I can’t handle shit anymore, like everything is just piling on top of me … when I self-harm everything comes at once. Guilt, regret, sadness, anger, hurt, basically a lot of emotions.” (Shaun).

In the above extract, Shaun describes how his acts of self-harm are typically preceded by an intense and unmanageable excess of negative emotions. Akin to how most participants reported emotional regulation difficulties, Shaun seemingly understood his self-harm as a way to reduce the intensity of his emotions and regain control during times in which he felt unable to cope. In the community, Shaun would mask his vulnerabilities through violence (see subtheme 2.3), enabling him to both self-regulate and maintain a positive identity, even when he felt vulnerable. However, in prison, self-harm fulfilled this function, offering Shaun a private means to reduce the intensity of his emotions whilst appearing in control to his peers on the wing and to avoid being perceived as ‘weak’.

Similar to Shaun, Ethan also described experiences of intense emotions whilst in custody and how he coped with them. Interestingly, Ethan construed punching his cell door as another form of self-harm:

“(Interviewer: Have you self-harmed more than once during this custodial sentence)? I punch my door init … You know if a screw pisses me off and they’re at my door, I’ll whack my door towards them, like I’ll hit my door, because if I don’t hit my door, I’ll hit them. And that’s why I try and like, like I try and take my anger out … Coz if I hit a person then I’m gonna get into trouble but if I hit a door then I’m not.” (Ethan).

Ethan storied this act as less destructive than interpersonal violence, with fewer consequences, whilst still allowing him to communicate, control, and reduce his intense feelings of anger. Although Ethan considers this act a form of self-harm, perhaps due to its injurious effects on his knuckles, it may be better construed as an act of violence (i.e., displacing his anger and violent intent toward the officers, and redirecting it towards an object). Whilst a different act and perhaps considered violence rather than self-harm within the spectrum of dual-harm behaviours, there is an interesting parallel between this behaviour and other self-harming behaviours. At the heart of this behaviour remains the need to express and release an intense emotion, whilst also communicating his frustration to the prison in a way that doesn’t compromise his position (“*if I hit a person then I’m gonna get into trouble but if I hit a door then I’m not.”*). Both Ethan’s and Shaun’s actions appear to be adapted to the environment. Ethan punches prison property rather than an officer to avoid negative consequences (e.g., segregation), whilst Shaun self-harms in private to maintain his social standing in the eyes of his peers. Interpreted in light of participants’ whole life stories, with more at stake in prison, these participants seemingly engage in these adapted methods to replace what may have been communicated or expressed through interpersonal violence in the community.

## 4 Discussion

This study was the first to explore the life stories of young adults in prison with a history of dual harm. An in-depth analysis of these life stories identified key experiences within participants’ lives, from childhood to present day, and explored how these were made sense of in relation to their dual harm behaviours. Participants described turbulent and intense relationships throughout their lives, seeking belonging and re-framed identities, and difficulties regulating their emotions. These themes were pertinent across participants’ lives and the ways in which they understood their behaviours.

Narratives that centred around early traumatic experiences were consistent across participants. These were severe, intense and often persistent in nature, including being the victim or witness of physical intrafamilial violence or neglect, which resonates with previous research (39, 40). These experiences provided a contextual backdrop to lives which were characterised by fractured relationships, a need to protect others, emotional dysregulation and fragile identities. Previous qualitative research with forensic populations has found that people who dual harm report multiple life adversities (11, 17), yet it is how people understand and respond to such adversities which is of interest in the current study. For example, following their experience of unstable relationships in childhood, participants sought connection and belonging from peers and romantic partners. Beliefs that such relationships were under strain seemed to underpin dual harm behaviours, whether that be to cope with the loss of a relationship or protect meaningful people in participants’ lives. Early trauma experiences contextualised and influenced participants’ later relational worlds, including their expectations of others, perceptions of threat, and strategies for managing emotional and interpersonal distress; all of which played important roles in their dual harm behaviours. Viewed through the lens of the power threat meaning framework (PTMF, 41), dual harm behaviours could be construed as “meaning-based threat responses” (p.9). That is, for some young men who dual harm, self-harm and violent behaviours may represent an understandable response to early adverse experiences, and functional attempts to manage perceived threats to connection, belonging or self-worth. This indicates that self-harm and violence should not be viewed purely as risk markers or maladaptive behaviours, but as understandable threat responses rooted in young men’s attempts to navigate life in the wake of challenging childhoods.

Another commonality was an inability to effectively regulate emotions following experiences such as loss and relationship breakdowns. This aligns with previous research which has linked deficits in emotional processing and regulation to dual harm (11, 40, 42, 43) and the increased risk of progressing from sole harm to dual harm (44). More specifically, self-harm and violence were described as tools used throughout participants’ lives to help them rebalance their emotional state to a tolerable level. This echoes theories suggesting that both behaviours can offer temporary relief from unwanted emotional arousal (45, 46). In this study, relief was often needed from intense anger or sadness, though overwhelming feelings of guilt and the need to self-punish were also apparent in participants’ narratives. It appeared that self-inflicted pain could reduce feelings of guilt whilst also being a method of atonement (47). Previous research indicated that in prison samples, self-harm may be more likely exhibited to reduce sadness or grief, whereas violence may reduce anger or aggression (11), though both behaviours were preceded by the same emotion among forensic mental health patients (17). In our findings, the environment in which the unwanted emotional state was experienced partially determined the exhibited behaviour. For example, whereas violence was commonly used to release anger and aggression in the community, self-harm in prison served a similar function whilst avoiding any punishments or consequences associated with violence; as previously reported by Power et al. (48). Therefore, whilst self-harm and violence shared the function of offering temporary relief from intense and unwanted emotional states, the environment played a crucial role in determining which behaviour was exhibited. These findings contextualise why people with violent histories may first engage in self-harm once imprisoned, and suggests interventions to support such individuals focus on the development of more effective emotion regulation strategies.

There were numerous narratives which described engaging in self-harm and violence to serve basic interpersonal needs (e.g., seeking a sense of belonging or respect, protecting the self or others, or signaling unmet needs to others). In line with previous research with forensic populations (11, 17), the decision between behaviours was partially determined by each behaviour’s perceived usefulness and accessibility. For instance, violence was predominantly used to communicate strength, acquire respect and gain belonging in the community, where, in prison, self-harm was used to signal unmet needs when other means of communication were considered ineffective. Engaging in harmful behaviours to feel listened to by prison staff is not uncommon in prisons (49) with some people self-harming to express frustrations with the system (50). Whilst violence in the community was framed as a necessary means to protect others, it was reframed as equally necessary to protect themselves in prison, allowing them to project an intensified, masculine version of themselves to survive hostile situations (21). According to Goffman’s (51) dramaturgical perspective, people choose how to present themselves and, depending on their environment, whether to reveal or conceal specific aspects of the self. An individual may present an aspect of the self to others (‘frontstage’ self) or keep it private (‘backstage’ self). Typically, both in prison and in the community, participants performed a ‘frontstage’ self which was centred around violence and projecting masculine ideals. By contrast, self-harm in prison was suggested to be a private ‘backstage’ self behaviour, simultaneously enabling individuals to regulate emotions, protect vulnerabilities, and maintain the ‘frontstage’ strong exterior to peers (51, 52). As such, there was an interplay between engaging in self-harm and violence to serve fundamental needs, whilst utilising each behaviour in distinct ways to coincide with how the men wanted to be perceived by others.

### 4.1 Implications for addressing dual harm in prisons

The consistent presence of traumatic early life experiences in this study suggest that a greater consideration of trauma in the histories of men in prison is needed. This includes up-skilling staff to be trauma-informed and developing trauma-responsive intervention practices to support people who dual harm in prison. To achieve the latter, aligning with the PTMF (41) assessment, formulation and intervention work should seek to understand (i) what the individual has been through in their lives, (ii) how individuals made sense of their life experiences, and (iii) how these experiences, the feelings they evoke and any associated meaning-making may be pertinent to their presenting behaviours.

It has been increasingly recognised that evidence-based talking therapies should be available to women in prison with a history of ACEs (53), and our findings strongly suggest that this should be extended to men. For example, therapy which focused on some of the key factors underpinning Dialectical Behavioural Therapy (e.g., developing emotion regulation skills, adaptive coping mechanisms and change techniques) helped forensic mental health patients to desist from dual harm (17). Moreover, compassion focussed approaches have shown positive outcomes in forensic populations such as reductions in perceptions of threat (54). As such, these interventions or similar talking therapies which prioritise emotion regulation and are delivered through a trauma-informed lens, could be used to support men who dual harm in prison.

Moreover, participants narrated fractured relationships throughout their lives. As a main source of relational interaction once in prison, perceiving prison staff as unhelpful or uncaring can undermine a person’s sense of safety (55). By contrast, increased psychological safety in prison has been found to reduce dual harm by removing the need for people to conceal vulnerabilities or deter potential threats (56). This, and the need to project masculine ideals in prison, suggests that interventions to reduce dual harm should challenge hegemonic masculinity and support people to adapt to prison life in ways which acknowledge contextual pressures. For example, peer support roles can help men in prison develop and perform positive masculinities (24). Therefore, cultural changes which seek to improve prison social climates, as well as interventions which challenge negative masculine norms and ideals, may be vital to reduce dual harm. With regards to the latter, further research is needed to develop an intervention to address dual harm, combining the principles discussed above, and explore its ability to improve outcomes for people in prison. By contrast, the cultural shift may be more complex to meaningfully achieve. Such cultural transformation may be especially complex, not least because prison social climates are notoriously hard to shift. These challenges are compounded by the fact that prison culture is shaped by, and cannot be divorced from, wider societal norms in which hegemonic masculinity remains deeply embedded. As such, addressing dual harm in prisons necessitates a multi- systemic approach, targeting the individuals displaying the behaviours and the wider contexts in which they are situated.

### 4.2 Limitations

This study included people in prison with a history of dual harm in the community, in prison, or both. Therefore, despite all participants having a history of dual harm, their experiences and understandings will have likely differed depending on the environment in which their dual harm behaviours were initiated. Future research is required to explore these groups separately (those who dual harmed only in prison, only in the community, or in both) to establish commonalities and differences in the meanings people attribute to their behaviours, and how this is influenced by the environment in which they are exhibited. Moreover, the definition of dual harm in this study was broad whereby people were eligible so long as they had engaged in at least one act of self-harm and violence. However, there may be differences between those who engage in repetitive and dynamic dual harm (i.e., they engage in both self-harm and violence repeatedly) and those who typically engage in one behaviour (i.e., self-harm or violence) who perhaps rarely engage in the second. Future research may wish to explore differences in the expression of self-harm and violence among these groups.

## 5 Conclusion

As the first study to qualitatively explore dual harm among young adult men in prison, this paper offers an important novel contribution to the emerging literature on dual harm. Findings illustrated that dual harm behaviours exhibited in prison cannot be meaningfully understood without attending to the impacts of early life adversities, sense-making of those experiences, and considering the adaptive functions that self-harm and violent behaviours serve. In this study, self-harm and violence were understood as serving conflicting but complementary functions and were underpinned by overarching themes of threat responses (e.g., a lack of safety or strained relationships), emotion regulation (e.g., suppressing or evoking emotions), interpersonal basic needs (e.g., to belong or seek revenge) and identity management (e.g., to present a masculine front or conceal one’s true self). Moreover, it became clear that environmental contexts played a key role in which behaviour was exhibited at any given time. Findings suggest that people who dual harm are adaptable and engage in behaviours which address their needs, but also those of the environment in which they are situated; and prison-based interventions must be responsive to this.

## Data Availability

The datasets presented in this article are not readily available because HMPPS restrictions. Requests to access the datasets should be directed to the corresponding author.
